# ﻿*Paraphlomishsiwenii* (Lamiaceae), a new species from the limestone area of Guangxi, China

**DOI:** 10.3897/phytokeys.212.91174

**Published:** 2022-11-03

**Authors:** Ya-Ping Chen, Jin-Fei Xiao, Chun-Lei Xiang, Xiong Li

**Affiliations:** 1 CAS Key Laboratory for Plant Diversity and Biogeography of East Asia, Kunming Institute of Botany, Chinese Academy of Sciences, Kunming, 650201, China Kunming Institute of Botany, Chinese Academy of Sciences Kunming China; 2 Guangxi Forestry Inventory and Planning Institute, Nanning, 530011, China Guangxi Forestry Inventory and Planning Institute Nanning China

**Keywords:** *Ajugoides*, karst, *Matsumurella*, nutlet, Paraphlomideae

## Abstract

The indumentum of nutlets is shown to be of phylogenetic importance in previous molecular phylogenetic studies of *Paraphlomis*, a genus of Lamiaceae with approximately 30 species distributed mainly in southern China and Southeast Asia. Nearly half the species of *Paraphlomis* are known from limestone areas. In this study, we described and illustrated a new species, *P.hsiwenii*, from the karst mountain forests in Guangxi Zhuang Autonomous Region, China. Our molecular phylogenetic analyses revealed that *P.hsiwenii* is recovered in a clade consisting of species with hairy nutlets. The new species is morphologically most similar to *P.pagantha* from the same clade, but they differ in the morphology of lamina bases, length of pedicels and calyces, as well as the morphology of upper corolla lips.

## ﻿Introduction

In a recently updated phylogenetic and taxonomic study of Lamioideae, the tribe Paraphlomideae was established to accommodate three genera, *Ajugoides* Makino, *Matsumurella* Makino, and *Paraphlomis* (Prain) Prain (Bendiksby et al. 2011). *Ajugoides* is a monotypic genus endemic to Japan and *Matsumurella* comprises five species distributed in East Asia (Harley et al. 2004; Bendiksby et al. 2011). As the largest genus of Paraphlomideae, *Paraphlomis* consists of ca. 30 species occurring mainly in China (especially in the south of the Yangtze River) and Southeast Asia (Wu and Li 1977; Li and Hedge 1994; Harley et al. 2004; Chen et al. 2021).

Morphologically, *Paraphlomis* can be distinguished by its herbaceous habit with stoloniferous stems and simple hairs, two to many-flowered verticillasters, actinomorphic and tubular to obconical calyces, 2-lipped (1/3) corollas, and apically truncate ovaries (Wu and Li 1977; Li and Hedge 1994; Harley et al. 2004; Chen et al. 2021). Two sections and five series were recognized in a previous infrageneric classification of *Paraphlomis* from China, divided based on the shape of calyx (tubular or obconical) and calyx teeth (e.g., conspicuous or inconspicuous, broadly triangular or subulate) (Li 1965; Wu and Li 1977). However, the most recent molecular phylogenetic study of *Paraphlomis* indicated that nutlet morphology (e.g., glabrous or hairy, obviously inflated or not) rather than the above-mentioned calyx characters is of phylogenetic value for the subdivision of the genus.

Species of *Paraphlomis* are mostly accustomed to shady and moist places in tropical and subtropical evergreen and mixed forests, and nearly half of the species are karst-adapted (Wu and Li 1977; Li and Hedge 1994; Zhang et al. 2020; Chen et al. 2022a). During our recent field investigations in the limestone area of Diding Natural Reserve in Guangxi Zhuang Autonomous Region, China, we found a putative new species of *Paraphlomis* which is characterized by hairy nutlets. We further confirmed its specific status as a new species and placement within the genus based on molecular phylogenetic and morphological evidence, and named it *P.hsiwenii* Y.P.Chen & XiongLi.

## ﻿Materials and methods

### ﻿Molecular phylogenetic analyses

We sampled a total of 34 accessions representing 19 species and four varieties/subspecies of *Paraphlomis* and two species of *Matsumurella* as the ingroups, and included two taxa, *Phlomisfruticosa* L. and Phlomoidesdentosavar.glabrescens (Danguy) C.L. Xiang & H. Peng from tribe Phlomideae as the outgroups. Only two accessions of the new species and one accession of *Paraphlomispagantha* Doan were newly sampled and sequenced here, while sequences of the remaining accessions were all retrieved from our previous studies (Chen et al. 2021, 2022a, b).

Total genomic DNA was extracted from silica-gel-dried leaf material using the modified CTAB method (Doyle and Doyle 1987). We selected five DNA markers for the phylogenetic reconstruction following previous studies (Chen et al. 2021, 2022a, b), i.e. the nuclear ribosomal internal and external transcribed spacers (ITS and ETS) and three plastid DNA regions (*rpl32-trnL*, *rps16*, and *trnL-trnF*). Primers used for the polymerase chain reaction (PCR) amplification and sequencing of the five regions, as well as the PCR mixtures and procedures, were the same as those described in Chen et al. (2021). Voucher information and GenBank accession numbers for all sequences are listed in Appendix 1.

Previous phylogenetic studies of *Paraphlomis* revealed significant topological incongruences between the nuclear and plastid trees (Chen et al. 2021, 2022a, b), therefore, we performed partitioned maximum likelihood (ML) and partitioned Bayesian inference (BI) analyses for the combined nuclear data set and combined plastid data set separately. Both the ML and BI analyses were conducted on the Cyberinfrastructure for Phylogenetic Research Science (CIPRES) Gateway (http://www.phylo.org/; Miller et al. 2010), using RAxML-HPC2 (Stamatakis 2014) and MrBayes (Ronquist et al. 2012), respectively. Detailed settings for the two analyses followed those described in Chen et al. (2019). TreeGraph 2 (Stover and Müller 2010) was employed to visualize and annotate the resulting trees.

### ﻿Taxonomic studies

Type specimens and protologues for all species of *Paraphlomis* were collated. Specimens of the genus from 21 public herbaria (BM, CDBI, E, GNNU, GXMI, HAST, HIB, IBK, IBSC, JIU, JJF, K, KUN, KYO, MW, NAS, PE, SM, SZ, TI, and WUK; abbreviations follow Thiers 2022) were also checked for the morphological comparison of *P.hsiwenii* with other species of *Paraphlomis*. Living plants of some species of the genus were observed and collected during our field investigation, and these specimens were further used for the morphological comparison of the new species. Other taxonomic and floristic literature related to *Paraphlomis* was reviewed, and the terminology used by Li and Hedge (1994) was adopted here for the morphological description of the new species.

## ﻿Results and discussion

**Figure 1.**
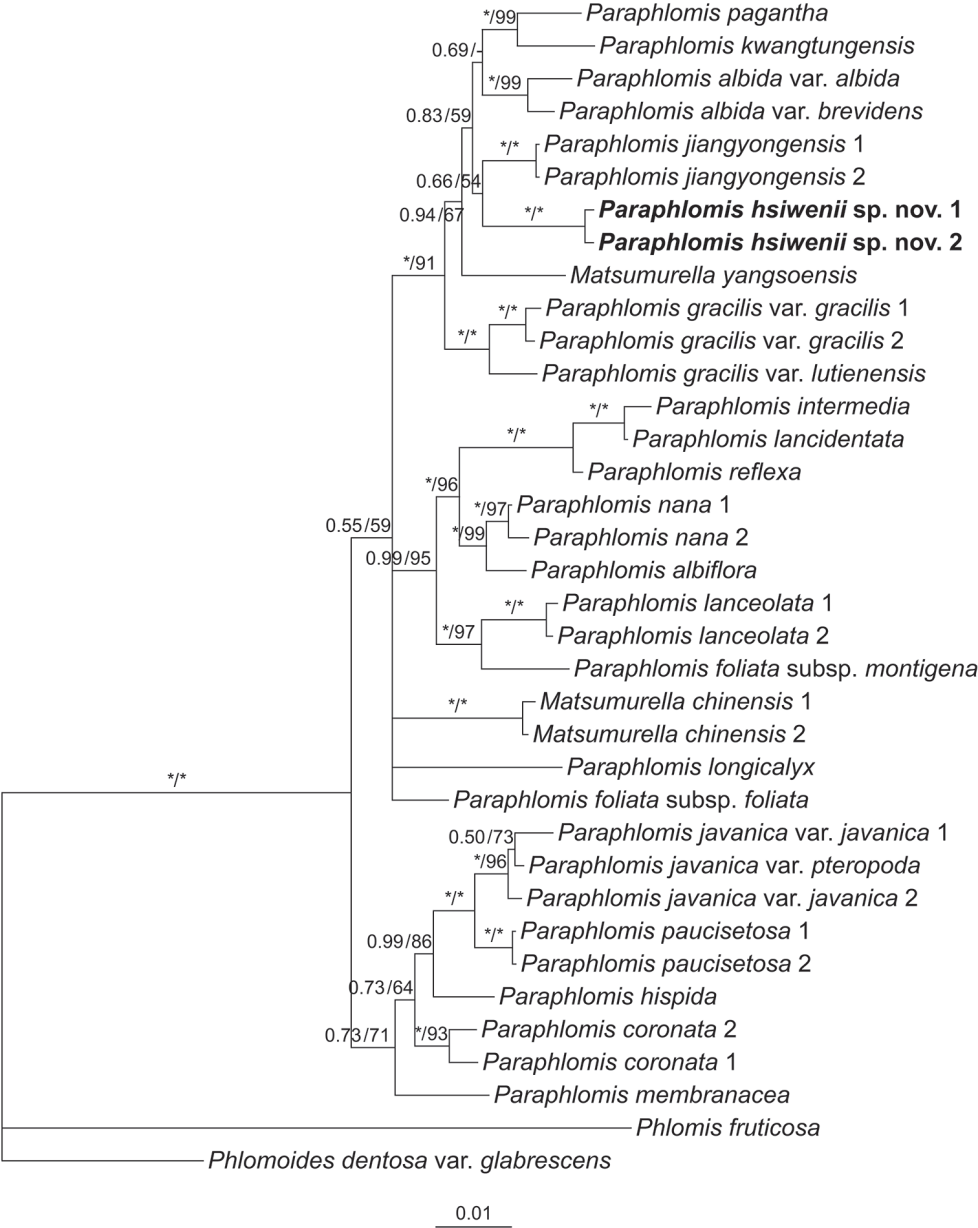
Bayesian 50% majority-rule consensus tree of *Paraphlomis* based on combined nuclear (ITS and ETS) data set. Support values ≥ 0.50 PP or 50% BS are displayed above the branches (an “*” indicates a support value = 1.00 PP or 100% BS and a “-” indicates a support value < 50% BS). Multiple accessions of the same species are numbered according to Appendix 1.

**Figure 2.**
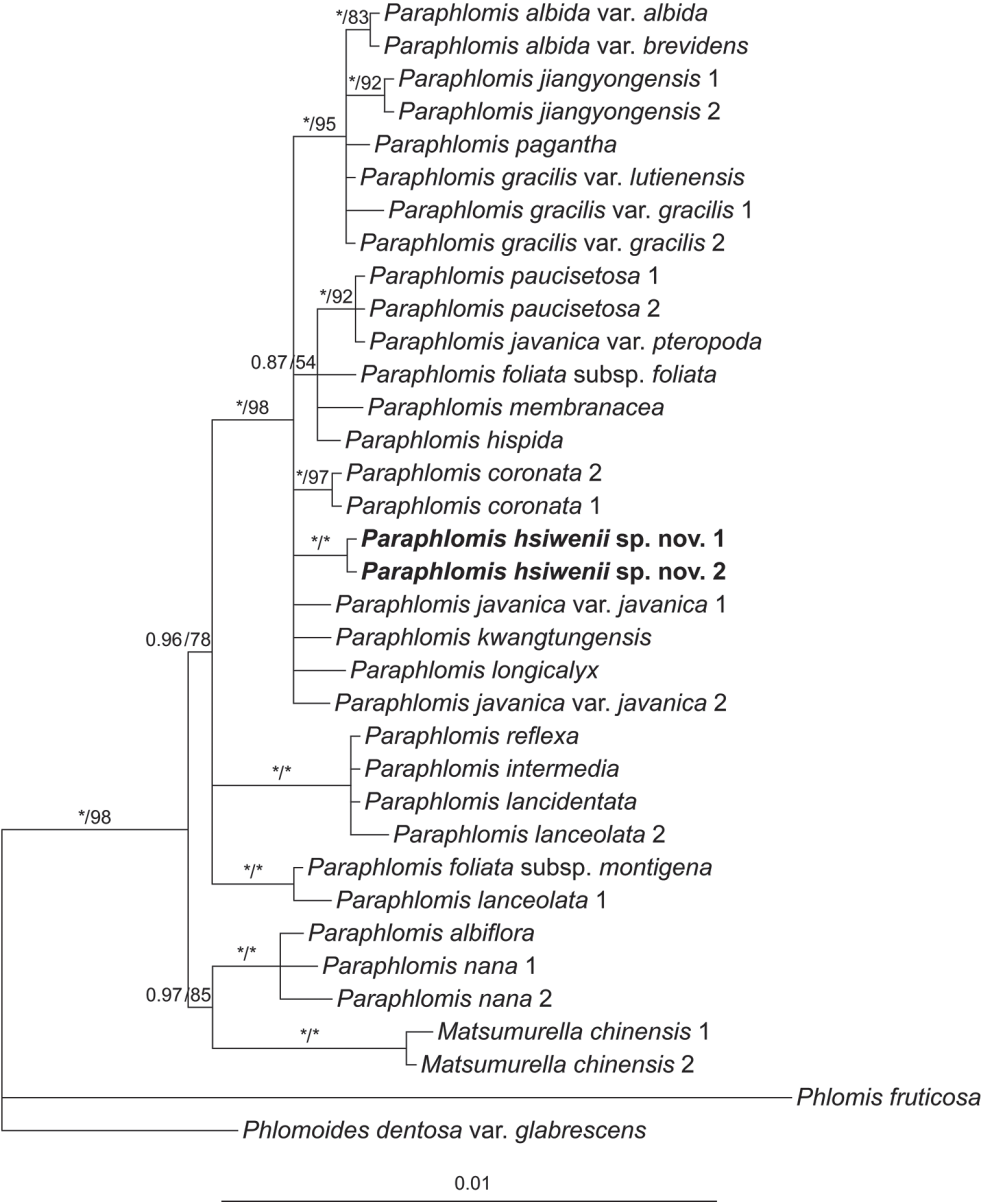
Bayesian 50% majority-rule consensus tree of *Paraphlomis* based on combined plastid (*rpl32-trnL*, *rps16*, and *trnL-trnF*) data set. Support values ≥ 0.50 PP or 50% BS are displayed above the branches (an “*” indicates a support value = 1.00 PP or 100% BS). Multiple accessions of the same species are numbered according to Appendix 1.

A total of 15 sequences (i.e., the five DNA regions of the two accessions of *P.hsiwenii* and one accession of *P.pagantha*) were newly generated in the present study. The aligned length of the combined nuclear data set and combined plastid data set was 1251 bp (808 bp for ITS, 443 bp for ETS) and 2479 bp (850 bp for *rpl32-trnL*, 812 bp for *rps16*, 817 bp for *trnL-trnF*), respectively. The topologies of the BI and ML trees were largely consistent with each other, but the BI trees are slightly better resolved. Thus, only the Bayesian 50% majority-rule consensus trees of the two combined data sets are presented, the posterior probabilities (PP) and Bootstrap support (BS) values being superimposed on the nodes (Figs 1, 2).

Both the nuclear and plastid data sets recovered species of *Matsumurella* within the *Paraphlomis* clade (Fig. 1: PP = 1.00/BS = 100%; Fig. 2: PP = 1.00/BS = 98%), indicating that neither of the two genera is monophyletic. As for relationships within the *Paraphlomis*-*Matsumurella* clade, some deep nodes in the nuclear tree (Fig. 1) and most shallow nodes in the plastid tree (Fig. 2) are poorly resolved, and topological conflicts between the two trees can be immediately recognized, especially at the placements of P.javanicavar.pteropoda D. Fang & K.J. Yan, *P.albiflora* (Hemsl.) Hand.-Mazz., and *P.nana* Y.P. Chen, C. Xiong & C.L. Xiang. The backbone topologies of *Paraphlomis* recovered in the present study are largely consistent with those of previous studies (Chen et al. 2021, 2022a, b), and the intergeneric relationships within Paraphlomideae and phylogenetic relationships within *Paraphlomis* have been discussed in Chen et al. (2021). Therefore, our following discussion will focus on the placement of the new species.

The two accessions of *P.hsiwenii* group together and form a strongly supported clade (Fig. 1: PP = 1.00/BS = 100%; Fig. 2: PP = 1.00/BS = 100%). In the plastid tree, relationships between *P.hsiwenii* and other species of *Paraphlomis* are not resolved (Fig. 2). In the nuclear tree, the new species is sister to *P.jiangyongensis* X.L. Yu & A. Liu but with weak support values (Fig. 1: PP = 0.66/BS = 54%). The two species are further placed within a robustly supported clade (Fig. 1: PP = 1.00/BS = 91%) together with *P.albida* Hand.-Mazz., *P.gracilis* (Hemsl.) Kudô, *P.kwangtungensis* C.Y. Wu & H.W. Li, *P.pagantha*, and *Matsumurellayangsoensis* (Y.Z. Sun) Bendiksby. This clade is corresponding to “Clade III” in Chen et al. (2021) and all its members have hairy nutlets/ovaries. The densely hispid and glandular nutlets of *P.hsiwenii* and its recovery within Clade III further support that nutlet morphology might be of phylogenetic significance for the infrageneric classification of *Paraphlomis* (Chen et al. 2021).

**Figure 3.**
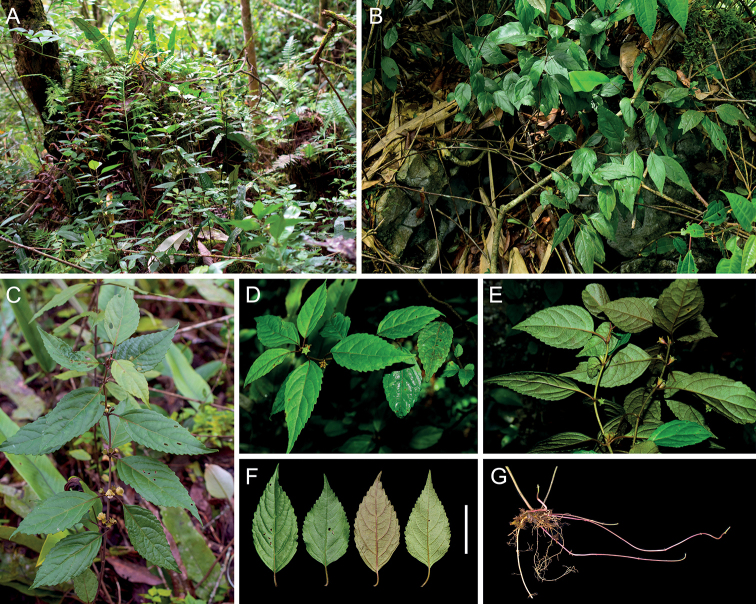
Morphology of *Paraphlomishsiwenii* from the type locality **A, B** habitat **C–E** habit **F** leaves **G** stolons. Scale bar: 5 cm (**A, C** photographed by Xiao-Lei Ma **B, D, E** photographed by Xiong Li **F** photographed by Ya-Ping Chen **G** photographed by Jin-Fei Xiao).

**Figure 4.**
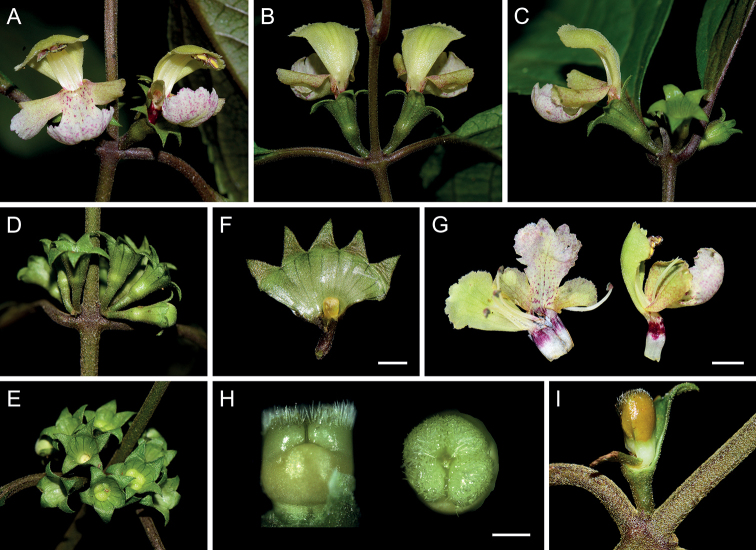
Floral traits of *Paraphlomishsiwenii*. **A** frontal view of flowers **B** dorsal view of flowers **C** lateral view of flowers **D** lateral view of calyces **E** frontal view of calyces **F** dissected calyx **G** dissected corolla and lateral view of corolla **H** lateral and frontal view of ovary **I** lateral view of nutlet. Scale bars: 2 mm (**F**); 4 mm (**G**); 500 μm (**H**) (**A–C, G, H** photographed by Jin-Fei Xiao **D–F** photographed by Ya-Ping Chen **I** photographed by Xiong Li).

**Figure 5.**
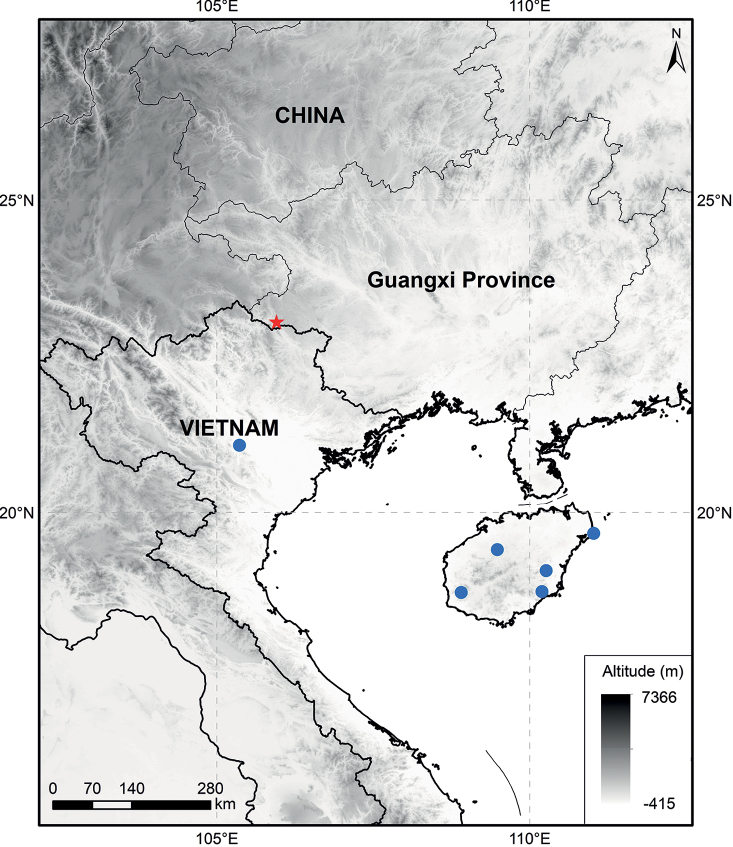
Distribution of *Paraphlomishsiwenii* (star) and *P.pagantha* (circle).

Morphologically, *P.hsiwenii* is most similar to *P.pagantha*, when comparing it with other species with hairy nutlets. For example, most species of Clade III are characterized by densely hispid or strigose stems and laminas, whereas both *P.hsiwenii* and *P.pagantha* have densely hispidulous stems and subglabrous to glabrous laminas (Fig. 3). They also share ovate laminas with serrate margins and triangular calyx teeth (Figs 3, 4). The two species differ mainly in the morphology of lamina bases, which are not decurrent in *P.hsiwenii* (Fig. 3) but obviously decurrent in *P.pagantha*. Another difference is that the length of pedicels and calyces is much longer in the new species (Fig. 4). Moreover, the upper corolla lips are ca. 6 mm wide with emarginate apices in *P.hsiwenii* (Fig. 4), but much narrower (ca. 3 mm wide) with entire apices in *P.pagantha*. Other morphological differences between the two species can be found in Table 1. Geographically, the new species is now only discovered from the limestone area in Diding Natural Reserve at the Sino-Vietnamese border, whereas *P.pagantha* usually grows in the evergreen forests in northern Vietnam and Hainan Province, China (Fig. 5). Notably, *P.pagantha* was treated as a synonym of *P.lancidentata* Y.Z. Sun by Suddee and Paton (2006). However, the nutlets of *P.lancidentata* are glabrous and the two species are recovered within different clades in the phylogenetic trees (Figs 1, 2).

**Table 1.** Morphological comparisons between *Paraphlomishsiwenii* and *P.pagantha*.

Characters	*P.hsiwenii*	*P.pagantha*
Lamina	Papery, ovate, base not decurrent, glabrous above	Papery to membranous, ovate to oblong, base decurrent, sparsely strigose above
Pedicel	2–3 mm long	Approximately 1 mm long
Calyx	Approximately 6 mm long, teeth ca. 2 mm long	Approximately 4 mm long, teeth ca. 1 mm long
Corolla	Tube purplish-red at upper part; upper lip greenish-yellow, ca. 8 × 6 mm, apex emarginate; lower lip ca. 8 × 10 mm, lateral lobes greenish-yellow	Tube with purplish-red spots at upper part; upper lip yellow to white, ca. 7 × 3 mm, apex entire; lower lip ca. 6 × 6 mm, lateral lobes yellow to white

### ﻿Taxonomic treatment

#### ﻿ Paraphlomis hsiwenii

Taxon classificationPlantaeLamialesLamiaceae

Y.P.Chen & XiongLi sp. nov.

4CC99465-333F-5831-8A9B-083D3FCA3BA0

urn:lsid:ipni.org:names:77307630-1

Figs 3 , 4

##### Type.

China, Guangxi, Jingxi City, Nanpo Town, Longting, Diding Natural Reserve, among shrubs in forests of limestone area, 23°3'29.55"N, 105°57'13.82"E, alt. 1181 m, 25 Jun 2022, J.F. Xiao & X.L. Ma XJF095 (holotype: KUN!; isotypes: K!, KUN!, MO!, PE!).

##### Diagnosis.

*Paraphlomishsiwenii* is morphologically most similar to *P.pagantha*, but differs in having laminas glabrous above (vs. sparsely strigose above), bases of laminas not decurrent (vs. decurrent), calyces ca. 6 mm long (vs. ca. 4 mm long) with teeth ca. 2 mm long (vs. ca. 1 mm long), and upper corolla lips emarginate at apex (vs. entire at apex).

Perennial herbs 50–120 cm tall, stoloniferous. Stems erect, simple or branched, obtusely 4-angled, densely retrorse hispidulous and glandular. Leaves opposite; lamina ovate, papery, 3–11 × 2.5–5 cm, apex acute to acuminate, margin serrate, base cuneate to broadly cuneate, adaxially green, glabrous, abaxially light green, purplish-green, or purple, densely glandular, lateral veins 3–5-paired; petioles 0.5–2 cm long, densely retrorse hispidulous and glandular. Verticillasters 2–14-flowered; bracteoles subulate, ca. 0.5 mm long, early deciduous; pedicels 2–3 mm long, densely retrorse hispidulous and glandular. Calyx green to yellowish-green, campanulate, ca. 6 mm long, densely hispidulous and glandular outside; teeth 5, subequal, triangular, reflexed, ca. 2 mm long, sparsely hispidulous inside, apex acute. Corolla ca. 1.4 cm long; tube ca. 6 mm long, ca. 1.5 mm wide, purplish-red at upper part; 2-lipped, upper lip oblong, yellowish-green, erect, concave, ca. 8 mm long, ca. 6 mm wide, sparsely pubescent outside, apex emarginate, lower lip ca. 8 mm long, ca. 1 cm wide, sparsely pubescent outside, 3-lobed, medium lob largest, white, dotted with purplish-red spots, subcircular, concave, apex emarginate, ca. 6 mm long, ca. 6 mm wide, lateral lobes ovate, yellowish-green, dotted with purplish-red spots, reflexed, ca. 4 mm long, ca. 2 mm wide. Stamens 4, straight, included, filaments hispid at base, anther cells 2, divergent. Style included, glabrous, apex subequally 2-lobed, lobes subulate. Ovary truncate at apex, densely hispid and glandular. Nutlets yellowish-brown, triquetrous-oblong, ca. 3.5 mm long, apex hispid and glandular.

##### Phenology.

Flowering from June to July, fruiting from July to August.

##### Distribution and habitat.

*Paraphlomishsiwenii* is currently only known from Diding Natural Reserve in Guangxi, China (Fig. 5). It occurs in shady places in evergreen broad-leaved forests or among shrubs in limestone mountains at an altitude of ca. 1200 m.

##### Etymology.

The new species is named after the Chinese taxonomist Hsi-Wen Li, who passed away in 2021 and had contributed tremendously to the taxonomy of Lamiaceae from China.

##### Chinese name

**(assigned here).** xī wén jiǎ cāo sū (锡文假糙苏).

##### Additional specimens examined.

China. Guangxi: Jingxi City, Nanpo Town, Longdingtun, Diding Natural Reserve, alt. 1231 m, 19 Aug 2020, W.H. Wu et al. DD426 (KUN).

##### Specimens of *P.pagantha* examined.

China. Hainan: Danzhou City, Shaposhan (Mt. Shamaoling), 29 Aug 1927, W.T. Tsang 672-16171 (IBSC0718446, IBSC0585106, PE00834801); Ledong County, Chang’e Village, Mt. Chang’eling, 16 Jun 1936, S.K. Lau 27154 (IBK00059945, IBSC0585103, KUN0274797, NAS00224460, PE00834800); Ledong County, Mt. Jianfengling, alt. 750 m, 16 Jun 1959, Z.F. Wei 122572 (IBSC0585104); Qionghai City, Huishan Natural Reserve, 21 Apr 2021, L.X. Yuan et al. s.n. (KUN); Wanning City, Xinglong Town, Langmingtian Village, Wutiaosang, 17 Jul 1935, F.C. How 73217 (IBK00059942, IBSC0585105, PE00834802); Wanning City, Xinglong Town, Mt. Niuguling, 16 Apr 1935, F.C. How 71953 (BM, IBK00059943, IBSC0585102); Wanning City, mountain behind Nanlin Nongchang, 5 May 1984, Z.X. Li et al. 1661 (IBSC0585107); Wenchang City, Tongguling Natural Reserve, 4 Aug 2021, L.X. Yuan et al. s.n. (KUN). VIETNAM. Tonkin (Hanoi): Mont. Bavi, Aug 1887, B. Balansa 2914 (Type: K000928198); Ba Vi, Son Tay, alt. 800 m, 14 Jun 1962, Ban 6893 (IBSC0616357).

## Supplementary Material

XML Treatment for Paraphlomis hsiwenii

## ﻿Appendix 1

**Table A1.** Sequence information for all samples used in present study. A “/” indicates a missing sequence. Herbarium abbreviations are listed after the vouchers. The accession numbers marked in bold represent sequences newly generated.

Taxon	Voucher	Country	ITS	ETS	*rpl32-trnL*	*rps16*	*trnL-trnF*
*Matsumurellachinensis* (Benth.) Bendiksby 1	Y. Yang OYY00316 (KUN)	Pingxiang, Jiangxi, China	MW602147	MW602117	MW602021	MW602053	MW602084
*Matsumurellachinensis* (Benth.) Bendiksby 2	Y. Yang OYY00131 (KUN)	Guilin, Guangxi, China	MW602148	MW602118	MW602022	MW602054	MW602085
*Matsumurellayangsoensis* (Y.Z. Sun) Bendiksby	L. Wu & W.B. Xu 10965 (IBK)	Yangshuo, Guangxi, China	MW602142	MW602112	/	/	/
ParaphlomisalbidaHand.-Mazz.var.albida	A. Liu et al. LK0841 (CSFI)	Ningyuan, Hunan, China	MW602124	MW602091	MW601996	MW602028	MW602060
Paraphlomisalbidavar.brevidens Hand.-Mazz.	Y.P. Chen EM312 (KUN)	Hezhou, Guangxi, China	MW602130	MW602098	MW602003	MW602035	MW602067
*Paraphlomisalbiflora* (Hemsl.) Hand.-Mazz.	C.M. Tan et al. 1806393 (JJF)	Jiujiang, Jiangxi, China	/	MW602101	MW602006	MW602038	MW602069
*Paraphlomiscoronata* (Vaniot) Y.P. Chen & C.L. Xiang 1	E.D. Liu et al. 3043 (KUN)	Emeishan, Sichuan, China	MW602137	MW602107	MW602012	MW602044	MW602075
*Paraphlomiscoronata* (Vaniot) Y.P. Chen & C.L. Xiang 2	C.L. Xiang 358 (KUN)	Jiangkou, Guizhou, China	MW602123	MW602090	MW601995	MW602027	MW602059
Paraphlomisfoliata(Dunn)C.Y. Wu & H.W. Lisubsp.foliata	S.P. Chen s.n. (KUN)	Jiangle, Fujian, China	/	MW602097	MW602002	MW602034	MW602066
Paraphlomisfoliatasubsp.montigena X.H. Guo & S.B. Zhou	Y.C. Dai s.n. (KUN)	Hangzhou, Zhejiang, China	OM836064	OM884453	OM884456	OM884459	OM884462
Paraphlomisgracilis(Hemsl.) Kudôvar.gracilis 1	A. Liu LK0931 (CSFI)	Changsha, Hunan, China	MW602134	MW602104	MW602009	MW602041	MW602072
Paraphlomisgracilis (Hemsl.) Kudôvar.gracilis 2	C.L. Xiang XCL1315 (KUN)	Chongqing, China	MW602141	MW602111	MW602016	MW602048	MW602079
Paraphlomisgracilisvar.lutienensis (Y.Z. Sun) C.Y. Wu	C.L. Xiang XCL881 (KUN)	Shibing, Guizhou, China	MW602131	MW602099	MW602004	MW602036	MW602068
*Paraphlomishispida* C.Y. Wu	X. Li LX200702 (GXF)	Napo, Guangxi, China	MW602132	MW602102	MW602007	MW602039	MW602070
***Paraphlomishsiwenii* Y.P.Chen & XiongLi 1**	**W.H. Wu et al. DD426 (KUN)**	**Jingxi, Guangxi, China**	**OP605346**	**OP609841**	**OP609848**	**OP609855**	**OP609862**
***Paraphlomishsiwenii* Y.P.Chen & XiongLi 2**	**W.H. Wu et al. DD426 (KUN)**	**Jingxi, Guangxi, China**	**OP605347**	**OP609842**	**OP609849**	**OP609856**	**OP609863**
*Paraphlomisintermedia* C.Y. Wu & H.W. Li	X. Zhong et al. ZX16823 (CSH)	Suichang, Zhejiang, China	MW602135	MW602105	MW602010	MW602042	MW602073
Paraphlomisjavanica(Blume)Prainvar.javanica 1	Y.P. Chen s.n. (KUN)	Kunming, Yunnan, China	MW602121	MW602088	MW601993	MW602025	MW602057
Paraphlomisjavanica(Blume)Prainvar.javanica 2	L.B. Jia et al. JLB0029 (KUN)	Maguan, Yunnan, China	MW602143	MW602113	MW602017	MW602049	MW602080
Paraphlomisjavanicavar.pteropoda D. Fang & K.J. Yan	X. Li 2020090501 (GXF)	Jingxi, Guangxi, China	MW602140	MW602110	MW602015	MW602047	MW602078
*Paraphlomisjiangyongensis* X.L. Yu & A. Liu 1	A. Liu et al. LK1104 (CSFI)	Jiangyong, Hunan, China	MW602128	MW602095	MW602000	MW602032	MW602064
*Paraphlomisjiangyongensis* X.L. Yu & A. Liu 2	A. Liu et al. LK1104 (CSFI)	Jiangyong, Hunan, China	MW602129	MW602096	MW602001	MW602033	MW602065
*Paraphlomiskwangtungensis* C.Y. Wu & H.W. Li	Y.P. Chen & Y. Zhao EM1391 (KUN)	Huaiji, Guangdong, China	MW602126	MW602093	MW601998	MW602030	MW602062
*Paraphlomislanceolata* Hand.-Mazz. 1	C.Z. Huang s.n. (KUN)	Guidong, Hunan, China	MW602145	MW602115	MW602019	MW602051	MW602082
*Paraphlomislanceolata* Hand.-Mazz. 2	A. Liu et al. LK0825 (CSFI)	Ningyuan, Hunan, China	MW602146	MW602116	MW602020	MW602052	MW602083
*Paraphlomislancidentata* Y.Z. Sun	X. Zhong et al. ZX16824 (CSH)	Suichang, Zhejiang, China	MW602136	MW602106	MW602011	MW602043	MW602074
*Paraphlomislongicalyx* Y.P. Chen & C.L. Xiang	Y.P. Chen et al. EM583 (KUN)	Huanjiang, Guangxi, China	OK104771	OK104774	OK104778	OK104780	OK104783
*Paraphlomismembranacea* C.Y. Wu & H.W. Li	M.S. Nuraliev 1057 (MW)	Thanh Son, Phu Tho, Vietnam	/	MW602100	MW602005	MW602037	/
*Paraphlomisnana* Y.P. Chen, C. Xiong & C.L. Xiang 1	C. Xiong XC21097 (KUN)	Chengkou, Chongqing, China	OM836062	OM884451	OM884454	OM884457	OM884460
*Paraphlomisnana* Y.P. Chen, C. Xiong & C.L. Xiang 2	C. Xiong & H.L. Zhou XC21126 (KUN)	Wushan, Chongqing, China	OM836063	OM884452	OM884455	OM884458	OM884461
***Paraphlomispagantha* Dunn**	**L.X. Yuan et al. s.n. (KUN)**	**Qionghai, Hainan, China**	**OP605345**	**OP609840**	**OP609847**	**OP609854**	**OP609861**
*Paraphlomispaucisetosa* C.Y. Wu 1	X.X. Zhu s.n. (KUN)	Malipo, Yunnan, China	MW602125	MW602092	MW601997	MW602029	MW602061
*Paraphlomispaucisetosa* C.Y. Wu 2	X. Li LX200704 (GXF)	Napo, Guangxi, China	MW602133	MW602103	MW602008	MW602040	MW602071
*Paraphlomisreflexa* C.Y. Wu & H.W. Li	Z.Z. Yang et al. s.n. (HIB)	Tongshan, Hubei, China	MW602122	MW602089	MW601994	MW602026	MW602058
*Phlomisfruticosa* L.	Y. Tong s.n. (KUN)	Shanghai, China (cultivated)	MW602119	MW602086	MW601991	MW602023	MW602055
Phlomoidesdentosavar.glabrescens (Danguy) C.L. Xiang & H. Peng	Y.P. Chen EM360 (KUN)	Beijing, China (cultivated)	MW602120	MW602087	MW601992	MW602024	MW602056

## References

[B1] Bendiksby M Thorbek L Scheen AC Charlotte L Olof R (2011) An updated phylogeny and classification of Lamiaceae subfamily Lamioideae. Taxon 60(2): 471–484. 10.1002/tax.602015

[B2] Chen YP Wilson TC Zhou YD Wang ZH Liu ED Peng H Xiang CL (2019) *Isodonhsiwenii* (Lamiaceae: Nepetoideae), a new species from Yunnan, China. Systematic Botany 44(4): 913–922. 10.1600/036364419X15710776741486

[B3] Chen YP Liu A Yu XL Xiang CL (2021) A preliminary phylogenetic study of *Paraphlomis* (Lamiaceae) based on molecular and morphological evidence. Plant Diversity 43(3): 206–215. 10.1016/j.pld.2021.03.002 34195505 PMC8233522

[B4] Chen YP Sun ZP Xiao JF Yan KJ Xiang CL (2022a) *Paraphlomislongicalyx* (Lamiaceae), a new species from the limestone area of Guangxi and Guizhou Provinces, southern China. Systematic Botany 47(1): 251–258. 10.1600/036364422X16442668423572

[B5] Chen YP Xiong C Zhou HL Chen F Xiang CL (2022b) *Paraphlomisnana* (Lamiaceae), a new species from Chongqing, China. Turkish Journal of Botany 46(2): 176–182. 10.55730/1300-008X.2680

[B6] Doyle JJ Doyle JD (1987) A rapid DNA isolation procedure for small quantities of fresh leaf tissue. Phytochemical Bulletin 19: 11–15.

[B7] Harley RM Atkins S Budantsev AL Cantino PD Conn BJ Grayer R Harley MM de Kok R Krestovskaja T Morales R Paton AJ Ryding O Upson T (2004) Labiatae. In: Kubitzki K Kadereit JW (Eds) The families and genera of vascular plants, vol.7. Springer, Berlin and Heidelberg, 167–275. 10.1007/978-3-642-18617-2_11

[B8] Li HW (1965) Revisio generis *Paraphlomis* Labiatarum Sinensium. Acta Phytotaxonomica Sinica 10: 57–74.

[B9] Li HW Hedge IC (1994) Lamiaceae. In: Wu CY Raven PH (Eds) Flora of China, vol.17. Science Press, Beijing and Missouri Botanical Garden Press, St. Louis, 269–291.

[B10] Miller MA Pfeiffer W Schwartz T (2010) Creating the CIPRES Science Gateway for inference of large phylogenetic trees. Proceedings of the Gateway Computing Environments Workshop (GCE). New Orleans, LA, 1–8. 10.1109/GCE.2010.5676129

[B11] Ronquist F Teslenko M van der Mark P Ayres DL Darling A Höhna S Larget B Liu L Suchard MA Huelsenbeck JP (2012) MrBayes 3.2: Efficient Bayesian phylogenetic inference and model choice across a large model space. Systematic Biology 61(3): 539–542. 10.1093/sysbio/sys029 22357727 PMC3329765

[B12] Stamatakis A (2014) RAxML version 8: A tool for phylogenetic analysis and post-analysis of large phylogenies. Bioinformatics 30(9): 1312–1313. 10.1093/bioinformatics/btu033 24451623 PMC3998144

[B13] Stover B Müller K (2010) TreeGraph 2: Combining and visualizing evidence from different phylogenetic analyses. BMC Bioinformatics 11(1): 1–9. 10.1186/1471-2105-11-7 20051126 PMC2806359

[B14] Suddee S Paton A (2006) Validation of Lamiaceae names. Kew Bulletin 61: 619–621.

[B15] Thiers B (2022) Index Herbariorum: a global directory of public herbaria and associated staff. New York Botanical Garden’s Virtual Herbarium. http://sweetgum.nybg.org/science/ih/ [Accessed Jul 2022]

[B16] Wu CY Li HW (1977) *Paraphlomis* Prain. In: Wu CY Li HW (Eds) Flora Reipublicae Popularis Sinicae, vol.65(2). Science Press, Beijing, 545–572.

[B17] Zhang RB Deng T Dou QL Wei RX He L Ma CB Zhao S Hu S (2020) *Paraphlomiskuankuoshuiensis* (Lamiaceae), a new species from the limestone areas of northern Guizhou, China. PhytoKeys 139: 13–20. 10.3897/phytokeys.139.47055 31997894 PMC6976690

